# The Story of the Insane from Year to Year

**Published:** 1895-10-26

**Authors:** 


### THE STORY OF THE INSANE FROM YEAR TO YEAR.

### ROYAL ASYLUM, PERTH.

The numbers in this asylum were practically stationary, being 110 at the beginning of the year and 112 at the close of it. There were 35 admissions, 12 recoveries, 8 cases discharged relieved, and 9 deaths. The percentage of recoveries on the admissions was 34·28, and that of the deaths on the average number resident was 8·93. Five of the deaths were due to cerebral and spinal diseases. Six of the admissions owed their insanity to drink, and 14 to hereditary influences. Several others were descended from families of neurotic tendency. Dr. Urquhart has the following sensible remarks on the causation of insanity: "An innate tendency to faulty cerebral action was developed into insanity in eight cases by the stress of mental worry, occasioned by business affairs, and it is noteworthy that the majority of these were men. On the other hand the stress of physical trouble has been more potent in the other sex, especially such disasters as arise in consequence of the strain thrown upon the system at critical times of life. Or, stating it in another way, the congenital *plus* mental causes were responsible for the greater proportion of male patients, the congenital *plus* physical causes for more female patients." This is really very well put. We learn from this report that one-half of the whole number of patients are allowed out on parole, and that only once during the year has it been necessary to withdraw this indulgence, which shows clearly enough that self-restraint is developed by granting liberty. It is as curious as it is humiliating to have to confess that this open-door system is almost confined to Scotland. We doubt whether there are three English or Irish county asylums where the open door system is in vogue; and although there may be some excuse for the non-development of freedom in those asylums where the proportion of attendants does not exceed one to ten patients, there is none whatever in our lunatic hospitals where the proportion is one to four or five; where, too, the patients are all picked cases. And yet we believe it would be susceptible of proof that our pauper lunatics have more freedom than the private patients. Even the Americans have begun to open their doors and pull down airing court walls while England is lagging behind. The commissioners say that "great ability is exhibited in the management of the institution." The weekly cost is £1 15s. 1d. per head; but 33 patients were kept at rates varying from £30 to £52 a year.

### SOUTH YORKSHIRE ASYLUM, WADSLEY.

Although smaller than several of the Middlesex and Lancashire asylums, it may be said truthfully that this one is twice too large. With 1,617 patients on the books, personal supervision must become so difficult that it verges on the impossible, and yet this blunder of building gigantic asylums is repeated again and again, regardless of the theoretical and practical objections which have been raised and never answered. There were 444 admissions, 163 recoveries, and 136 deaths during the year 1894. These numbers show that the recoveries stand at the fair percentage of 36·7 calculated on the admissions, and the deaths at 8·5 on the average number resident. Of the deaths 75 were caused by cerebral and spinal diseases, 17 to consumption, and 1 to typhoid fever. Not fewer than 75 of the cases admitted were driven mad by drink, 208 by adverse circumstances and family trouble, and 85 by hereditary influence. Instruction of the attendants and nurses is carried on in this asylum by the assistant medical officers, Dr. Ruxton and Dr. Macpherson, and four nurses and seven attendants have passed the examination of the medico-psychological examinations. Dr. Kay thinks these successful ones should have some suitable acknowledgment, and we agree with him, for although virtue is its own reward, and the knowledge obtained is useful to the individual himself, it cannot be denied that a little stimulus is desirable. We hope, therefore, the South Wadsley Committee will act on Dr. Kay's suggestion, and we venture to advise an increase of wages, which is perfectly justifiable, because the patients are better looked after, and to this might be added medals or badges, for undoubtedly most women and all men like to be decorated. We notice there is a balance of £920 in favour of the farm, and further, that £220 was transferred from the private patients' profit account to buy cows. We fear this is not in harmony with the Act, and we doubt whether a West Riding auditor can be induced to believe that ten cows are improvements or additions to the building; but there is no knowing what an auditor will object to or what he will pass. The weekly rate is 9s. 5¾d.

